# Late prosthetic valve endocarditis with *Mycobacterium tuberculosis* after the Bentall procedure

**DOI:** 10.1186/s12941-019-0314-0

**Published:** 2019-03-28

**Authors:** Qianqian Liu, Jialin Jin, Lingyun Shao, Shanshan Weng, Ju Zhou, Feng Li, Wenhong Zhang, Xinhua Weng, Yan Gao

**Affiliations:** 1 Department of Infectious Diseases, Huashan Hospital, Fudan University, 12 Wulumuqi Zhong Road, Shanghai, 200040 China; 2 Department of Cardiac Surgery, Shanghai Sixth People’s Hospital, Shanghai, China; 3 Key Laboratory of Medical Molecular Virology, Ministry of Education and Health, Shanghai Medical College and Institutes of Biomedical Science, Fudan University, Shanghai, 200032 China

**Keywords:** *Mycobacterium tuberculosis*, Prosthetic valve endocarditis, Mechanical valve

## Abstract

**Background:**

Prosthetic valve endocarditis (PVE) is a rare but severe complication of valve replacement surgery, with an incidence rate of 0.3–1.2% per patient-year. At present, staphylococci are the predominant causative microorganism of PVE. Herein, we report a confirmed case of late PVE in a mechanical aortic valve caused by *Mycobacterium tuberculosis*.

**Case presentation:**

A 32-year-old immunocompetent man with recurrent fever and 5-kg weight loss had a history of having undergone the Bentall procedure due to congenital heart disease. Nine years after the operation, he developed a paravalvular abscess in the mechanical aortic valve, presented with evidence of pulmonary tuberculosis on CT scan and was diagnosed with tuberculous endocarditis. This case report highlights a rare and non-negligible example of tuberculous endocarditis involving a mechanical valve.

**Conclusions:**

Tuberculous PVE should be considered in patients with a history of valve replacement, recurrent fever, unexplained weight loss, pulmonary tuberculosis and meaningful valvular findings on echocardiogram.

## Background

Prosthetic valve endocarditis (PVE), a rare but severe complication of valve replacement surgery, with an incidence rate of 0.3–1.2% per patient-year, accounts for 20% of all cases of infective endocarditis (IE) and has a high mortality rate. At present, staphylococci are the predominant causative microorganism of PVE [1, 2]. We report a confirmed case of late PVE (> 1 year post-surgery) in mechanical aortic valve caused by *Mycobacterium tuberculosis* in a patient who previously underwent a Bentall procedure.

## Case presentation

A 32-year-old man was admitted to our hospital on June 5, 2018, with a 2-month history of recurrent fever and the loss of 5 kg of weight. Two months prior, he suffered chills after eating kebabs, followed by a fever (up to 39 °C), accompanied by a headache and dizziness. He received anti-infective and anti-inflammatory therapy for a pulmonary infection at the local hospital. However, he still had a high fever with fatigue. On April 23, the serum agglutination test for *Brucella* was positive, with a titer of 1:400. Both the blood and bone marrow cultures were negative, and after he was diagnosed with brucellosis, therapy with doxycycline, moxifloxacin, and streptomycin was initiated. After 1 month of regular anti-brucellosis therapy, although the peak temperature had dropped to 38 °C, he still had the fever and aggravated anaemia. Due to the negative repeat test for *Brucella*, the regimen was adjusted to isoniazid, levofloxacin, streptomycin, rifampicin and dexamethasone anti-tuberculosis therapy. He continued to experience the recurrent fever until admission to our hospital on June 5.

In March 2009, he had undergone a Bentall procedure and ventricular septal repair due to congenital heart disease and was found to be positive for the hepatitis B surface antigen but did not receive antiviral therapy. The patient was immunocompetent, serologically negative for human immunodeficiency virus (HIV) and denied any history of contact with cattle and sheep.

**Fig. 1:**
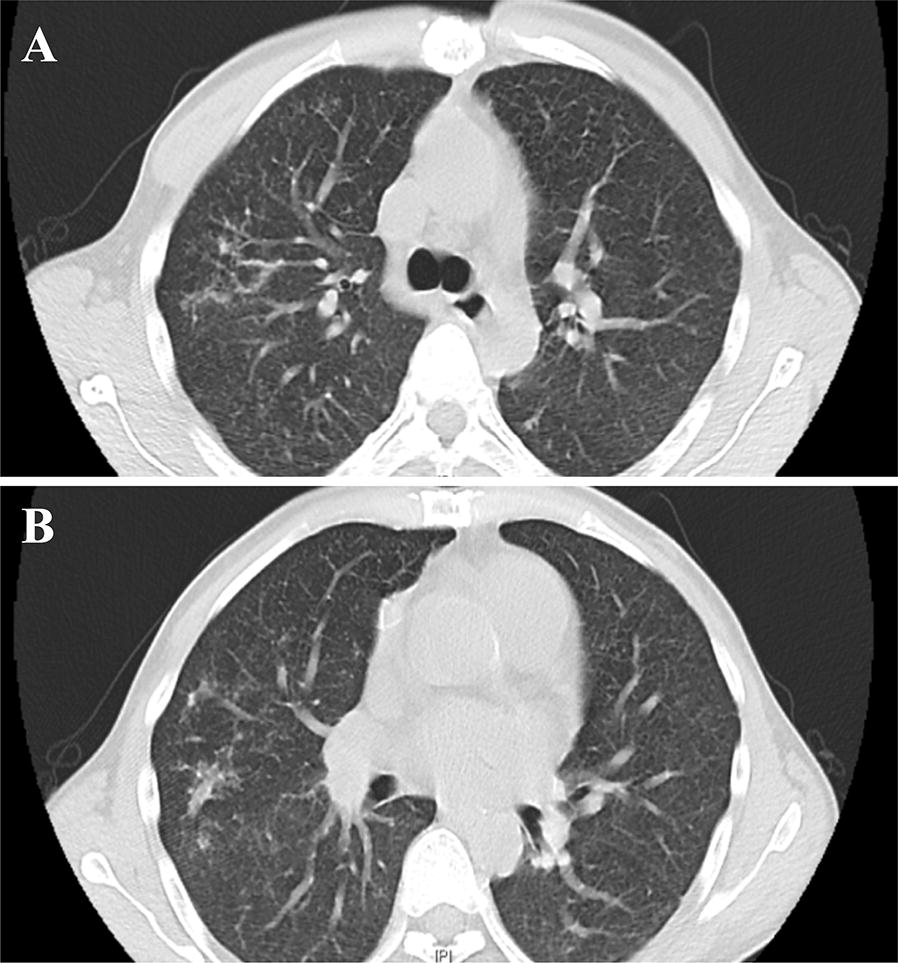
Axial computed tomography (CT) scan of the chest showing thickened lung markings with patchy shadows in both the upper lobes (**A**) and the lower lobes (**B**)

**Fig. 2:**
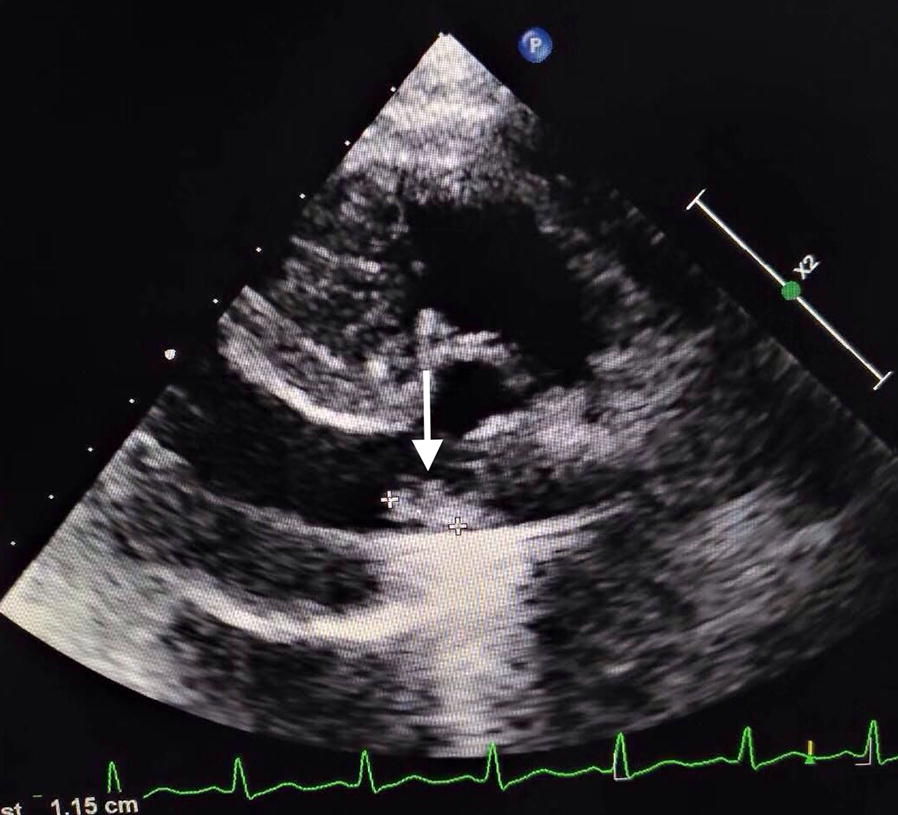
Transoesophageal echocardiogram showing a paravalvular abscess on the prosthetic aortic valve (arrow)

At admission, his body temperature was 37.5 °C, his heart rate was 78 beats/min, his respiratory rate was 18 breaths/min, and his blood pressure was 120/66 mmHg. On physical examination, he was found to be anaemic. His abnormal laboratory findings were as follows: aggravated normochromic anaemia (haemoglobin: 71 g/mL), elevated levels of inflammatory markers (C-reactive protein: 43.1 mg/L; procalcitonin: 0.38 ng/mL), acute renal insufficiency (blood urea nitrogen: 23.1 mmol/L; creatinine: 343 μmol/L) and hypoproteinemia (albumin: 30 g/L) with normal levels of aminotransferase (Table 1). An interferon-gamma release assay (IGRA) for tuberculosis was positive. Furthermore, next-generation sequencing (NGS) detected *M. tuberculosis* complex from two blood samples. However, the Xpert MTB assay and acid-fast bacilli smear for sputum samples were both negative. The blood cultures remained negative. A computed tomography (CT) scan of the chest suggested bilateral pneumonia (Fig. 1). Abdominal ultrasound revealed enlargement of his liver, spleen (169 × 65 mm) and kidney (left: 145 × 67 mm; right: 146 × 52 mm). Echocardiography showed mechanical prosthetic aortic valve paravalvular abscess without vegetation formation (Fig. 2). Then, the patient was empirically administered fosfomycin and daptomycin as a treatment for gram-positive bacterial infection, but his body temperature remained high (up to 38.4 °C). Thus, the regimen was switched to anti-tuberculosis drugs (isoniazid 0.3 g once daily, ethambutol 0.75 g once daily, moxifloxacin 0.4 g once daily and linezolid 0.6 g once daily); however, his temperature remained between 37 and 38.5 °C.

**Table 1:** Laboratory examinations

Clinical parameters	Reference range	On admission	Postoperative
RBC (× 10^12^/L)	4.3–5.8	2.55	3.47
WBC (× 10^9^/L)	3.5–9.5	4.36	5.99
Neutrophil (%)	40–75	80.9	48.3
Haemoglobin (g/L)	130–175	71	105
Platelet (× 10^9^/L)	125–350	126	180
INR	0.92–1.15	1.23	1.54
ESR (mm/h)	≤ 15	14	30
CRP (mg/L)	< 8.2	43.1	–
PCT (ng/mL)	≤ 0.05	0.38	0.19
BUN (mmol/L)	2.5–7.0	23.1	8.16
Creatinine (μmol/L)	50–130	343	140
ALT (U/L)	9–50	29	9
AST (U/L)	15–40	36	22
Albumin (g/L)	40–55	30	43.7

*RBC* red blood cell, *WBC* white blood cell, *INR* international normalized ratio, *ESR* erythrocyte sedimentation rate, *CRP* C-reactive protein, *PCT* procalcitonin, *BNP* brain natriuretic peptide, *BUN* blood urea nitrogen, *ALT* alanine aminotransferase, *AST* aspartate aminotransferase

Then, he was transferred to the Department of Cardiac Surgery, Shanghai Sixth People’s hospital for a second surgery on June 25. Surprisingly, NGS detected a large number of *M. tuberculosis* complex sequences in intraoperative samples of the aortic root abscesses. The patient had postoperatively intermittent fever, mostly peaking in the afternoon. After 2 weeks of combined anti-tuberculosis treatment with linezolid (0.6 g once daily), rifampicin (0.3 g twice daily), pyrazinamide (0.5 g three times daily), ethambutol (0.75 g once daily), isoniazid (0.3 g once daily) and moxifloxacin (0.6 g once daily), his body temperature returned to normal, and his laboratory parameters improved (Table 1); finally, he was discharged. On July 31, the culture of the aortic valve intraoperative scrapings was found to be positive for *M. tuberculosis*, which further confirmed the diagnosis of tuberculous PVE. To date, he continues to receive oral anti-tuberculosis treatment.

## Discussion and conclusions

In this patient, the lack of specific symptoms increased the difficulty of the diagnosis, and his diagnosis of tuberculous PVE was finally confirmed by the results of the valve culture 1 month after his second operation. We speculated that the prosthesis infection originated from pulmonary lesions based on the findings on the chest CT, positive IGRA result and detection of *M. tuberculosis* in two blood samples using NGS, despite the negative blood cultures. Although the initially positive serum agglutination test for *Brucella*, the history of eating kebabs and the negative results of the blood and bone marrow cultures resulted in the diagnosis of brucellosis, the repeated serum agglutination test was negative, and more importantly, his clinical symptoms had not improved after 1 month of anti-brucellosis therapy. Therefore, brucellosis was ruled out, and other weakly virulent pathogens that can cause atypical clinical signs of IE needed to be considered.

Tuberculous endocarditis (TBE) is rare, and the infection sites in the majority of the previously reported TBE cases were native valves [3]. However, the infection of prostheses, especially mechanical prostheses, caused by *M. tuberculosis* has rarely been reported [4]. Here, we reported an extremely rare case of TBE involving a mechanical prosthetic aortic valve accompanied by paravalvular abscess, which was confirmed by valve culture that was positive for *M. tuberculosis*. According to the timing of presentation after valve surgery, PVE is defined as early (< 1 year) and late (> 1 year). Significant differences exist in the microbiology of early and late PVE. Early PVE generally originates from perioperative contamination or infections associated with the central venous catheter, which are mainly caused by staphylococci, fungi, and Gram-negative bacilli. In contrast, late PVE is often the result of prosthesis seeding due to transient bacteraemia from a distant site, which is mostly the result of community-acquired infections [4]. Our patient underwent a Bentall procedure 9 years ago, and this procedure is complicated by infection in 1.4% of cases, most commonly within 5 years after the procedure [5]. Currently, IE occurs more than 1 year after Bentall surgery in 65% of patients and within 4 weeks after surgery in only 20% of patients. More importantly, the most common causative pathogen is *Staphylococcus aureus* (45%) [6]. To the best of our knowledge, this is the first report of TBE involving a mechanical valve prosthesis after a Bentall procedure.

Several cases of TBE were reported between 1990 and 2017, and the characteristics of these patients are summarized in Table 2. The median age of TBE patients was 42.5 years, with a male/female ratio of 3:2. Of note, the infection sites among these TBE patients were all native valves, and most (7/10, 70%) were only single valve infections, with the exception of 3 cases involving multiple valves. In case 8, the patient used intravenous drugs, was infected with HIV and was diagnosed with TBE by blood cultures that were positive for *M. tuberculosis*. However, the other cases were free of HIV infection. Among all cases, the most critical management strategy was surgical intervention combined with anti-TB therapy. Additionally, the duration of anti-TB treatment in most cases was prolonged to 9 months or even to 12 months.

**Table 2:** Characteristics of reported patients with tuberculous endocarditis

Patient	Year	Age/sex	Valve	Diagnosis	Surgery	Anti-TB (months)	Outcome	References
1	2017	70/F	Mitral and aortic valves; native	TTE	Yes	9	Recovery	[8]
2	2016	1/F	Mitral valve; native	Vegetation AFB staining + PCR	Yes	12	Recovery	[9]
3	2015	21/F	Aortic valve; native	Blood culture	Yes	12	Recovery	[10]
4	2015	50/M	Mitral and aortic valves; native	TTE + sputum culture	Yes	9	Recovery	[11]
5	2012	17/F	Mitral, aortic and tricuspid valves; native	Histopathology of vegetation	Yes	Unspecified	Recovery	[12]
6	2010	30/M	Mitral valve; native	Vegetation culture	Yes	12	Recovery	[13]
7	2007	63/M	Aortic valve; native	Histopathology of aortic cusps	Yes	9	Recovery	[14]
8	2002	35/M	Tricuspid valve; native	Blood culture	Yes	6	Recovery	[15]
9	1998	64/M	Mitral valve; native	Vegetation culture	Yes	12	Recovery	[16]
10	1990	78/M	Aortic valve; native	echocardiogram + multiple specimens culture	Yes	12	Recovery	[17]

*TB* tuberculosis, *M* male, *F* female, *TTE* transthoracic echocardiogram, *AFB* acid-fast bacilli

Despite the low incidence of TBE in valvular prostheses, there were still several cases reported before 1990, and most occurred in bio-prosthetic valves including homograft and porcine valves [7]. In 1979, the first case of TBE affecting a mitral valve prosthesis 5 months post-operation was reported; the patient was diagnosed after the isolation of acid-fast bacilli (AFB) from the mitral valve prosthesis during the autopsy. Subsequently, two patients with miliary TB after prosthetic valve replacement survived less than 1 year.

This case suggests that tuberculous PVE should be taken into account in patients with a history of valve replacement, recurrent fever, unexplained weight loss, pulmonary TB and meaningful valvular findings (including vegetation or abscess) on echocardiogram. Rapid diagnosis facilitates the early administration of clinical treatment and decreases mortality in patients with TBE.

## Abbreviations

PVE: prosthetic valve endocarditis
IE: infective endocarditis
TBE: tuberculous endocarditis
AFB: acid-fast bacilli
IGRA: interferon-gamma release assay
